# The Lost and Found: Unraveling the Functions of Orphan Genes

**DOI:** 10.3390/jdb11020027

**Published:** 2023-06-13

**Authors:** Ali Zeeshan Fakhar, Jinbao Liu, Karolina M. Pajerowska-Mukhtar, M. Shahid Mukhtar

**Affiliations:** Department of Biology, University of Alabama at Birmingham, 1300 University Blvd., Birmingham, AL 35294, USA

**Keywords:** ORFans, Orphan Genes, functional characterization, dark transcriptomics, evolution, sequencing, high-throughput

## Abstract

Orphan Genes (OGs) are a mysterious class of genes that have recently gained significant attention. Despite lacking a clear evolutionary history, they are found in nearly all living organisms, from bacteria to humans, and they play important roles in diverse biological processes. The discovery of OGs was first made through comparative genomics followed by the identification of unique genes across different species. OGs tend to be more prevalent in species with larger genomes, such as plants and animals, and their evolutionary origins remain unclear but potentially arise from gene duplication, horizontal gene transfer (HGT), or de novo origination. Although their precise function is not well understood, OGs have been implicated in crucial biological processes such as development, metabolism, and stress responses. To better understand their significance, researchers are using a variety of approaches, including transcriptomics, functional genomics, and molecular biology. This review offers a comprehensive overview of the current knowledge of OGs in all domains of life, highlighting the possible role of dark transcriptomics in their evolution. More research is needed to fully comprehend the role of OGs in biology and their impact on various biological processes.

## 1. Introduction

The origin of genes and their role in evolution have been topics of interest for many years. In the -omics era, substantial evidence supports the theory that there was only one time in evolution in which all building blocks of genes originated and were subsequently shuffled and mixed to create novel configurations, perhaps aided by transcriptional and translational “noise” facilitating the emergence of new genes over time. However, recent studies on Orphan Open Reading Frames (ORFans), also known as Orphan Genes (OGs), suggest a different scenario. OGs constitute a unique class of genes that are thought to play a critical role in evolution and speciation. They are defined as genes lacking detectable homologs in other species, likely to be derived from a unique ancestral gene [1]. Typically, OGs encode short proteins with a high non-synonymous rate of substitution, and their functions are still largely unknown due to a lack of phylogenetic conservation [2,3]. OGs exhibit a narrow phylogenetic distribution, with every species documented to possess as much as 30% of OGs out of all gene catalogs [4]. 

OGs have a shorter origination time than non-Orphan Genes (non-OGs) [5,6]. They are characterized by fewer exons, and, at the protein level, shorter lengths and higher isoelectric points [1,4,7]. For instance, in the *Cucurbitaceae* family, OGs exhibited significantly shorter protein lengths in eight species. Moreover, comparative studies also revealed that OGs are characterized by fewer exons [8] and higher isoelectric points than non-OGs [9,10]. Changes in the isoelectric point are essential indicators of altered protein function and are often considered a unique adaptive characteristic under variable environmental conditions [10,11]. The GC content of OGs is heterogeneously distributed among species, with some having much higher GC content in OGs, and others showing significantly lower GC content [10,12]. Overall, OGs are a unique characteristic of any species and possess several distinguishing features that set them apart from non-OGs. They provide a vast reservoir of functional proteins with a tremendous rate of evolution, making it nearly impossible to trace any homological features. Recent studies have shown that homology detection failure may explain many OGs, and more sensitive synteny-based homology searches have successfully found previously undetected OGs [13].

OGs are important in evolution and speciation because they provide a mechanism for the production of novel genes and functions [7]. As such, they are thought to play a critical role in the evolution of species, as they allow organisms to respond to changes in their environment and develop new adaptations [14]. In many cases, the evolution of new functions through the creation of new genes is a driving force behind the divergence of species and the development of new species [1,15]. OGs have diverse functions, ranging from basic metabolic functions to complex regulatory processes (Table 1). For example, some OGs are involved in the regulation of development and growth, while others play a role in the response to environmental stresses [10]. Recent studies in *Caenorhabditis elegans* (*C. elegans*) have shown that OGs are involved in the regulation of developmental processes, such as the formation of sensory neurons and the regulation of muscle development [7]. Similarly, in mammals, OGs have been implicated in various diseases, including cancer and developmental disorders [16]. Additionally, some OGs contribute to the evolution of species-specific adaptations, such as the development of novel traits or adaptations to new environments [17]. For example, Hydra has a unique set of OGs that have a distinct role in phylum-specific morphological diversities and their innate defense systems [18,19]. This review aims to provide a comprehensive overview of the current understanding of OGs, including their origin, evolution, and function. It will examine the mechanisms that contribute to the formation of OGs, as well as the current state of knowledge regarding their distribution and functional significance across a wide range of species, including plants, animals, insects, humans, viruses, and prokaryotes. Furthermore, we suggest a systems-level approach for the identification and characterization of OGs through an ortholog analysis. Additionally, the present study provides a comprehensive analysis of the current difficulties, potential approaches, and potential future directions in the functional characterization of OGs.

## 2. Mechanisms of OG Origination 

The discovery of OGs is relatively recent and was made possible by the availability of complete genome sequences and high-throughput sequencing technologies, which have allowed researchers to identify and characterize OGs in various organisms, ranging from bacteria to higher eukaryotes [50,51]. The evidence suggests that the majority of these genes are the result of horizontal gene transfer, a process in which genes are acquired from distantly related organisms through mechanisms such as bacteriophage infection or conjugation [52,53]. 

Alternatively, a commonly held theory is that OGs can arise through a process of duplication and divergence that involves the duplication of a gene, followed by rapid evolution, which results in the loss of all visible similarities to the original gene [54]. This scenario has limitations, including explaining how natural selection would isolate one of a duplicated pair for further evolution while maintaining the other for the preservation of the ancestral function [55,56]. Additionally, a high number of mutations are required for a protein to diverge to the point of no longer being identifiable by Basic Local Alignment Search Tool (BLAST), which is an uncommon occurrence, as many genes have functional domains that are resistant to mutations [57]. Modifications to the duplication–divergence hypothesis have been proposed to address these limitations. For example, the original open reading frame (ORF) could be altered due to a rearrangement or transposon insertion, allowing for further evolution [58,59]. Alternatively, the original ORF could become inactive after duplication, leading to the utilization of a new reading frame and the production of a completely new protein [60]. However, no incidence of a new protein being produced in this manner has been documented to date. Moreover, parasitism, specifically the interactions in molecules between the bacterial host and the phage, is also one of the proposed modules suggesting how new genes are acquired/born [52]. 

De novo gene origination is another mechanism for the genesis of OGs [61]. It involves the creation of a new gene from non-coding DNA sequences, typically through the rearrangement of existing genomic regions or the formation of new transcriptional units. This process is believed to play a significant role in the evolution of OGs, particularly in the evolution of complex multicellular organisms such as mammals [59,62]. Another proposed phenomenon related to OG formation is the emergence of OGs from non-coding regions of the genome or through rapid divergence of the coding sequence (CDS) of an existing gene [13]. This divergence can occur due to a partial pseudogenization process, where the original gene becomes non-functional and evolves into an OG. It has been observed that over 80% of OGs are absent in newly automated genomes [11]. Consequently, the origination of OGs is a multifaceted and ever-changing phenomenon that is influenced by numerous mechanisms. The significance of these genes in the evolution of new traits and the control of crucial organismal functions have made them a crucial focus of study for scientists in the fields of evolution, genetics, and biochemistry. Factors such as mutation, selection, population size, neofunctionalization, and subfunctionalization influence the evolution of OGs [63]. Moreover, mutations in OGs may result in the creation of new functional elements or the loss of function of existing elements [44]. Selection also plays a role in preserving beneficial mutations, while population size may influence the frequency of mutations and the likelihood of genetic drift. 

## 3. Identification of OGs

**Figure 1:**
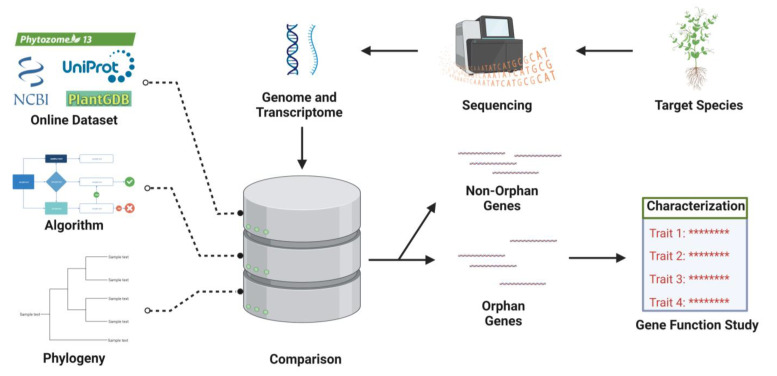
Schematics of Orphan Genes (OG) identification. Sequencing is used to acquire genomic or transcriptomic data of target species. The comprehensive analysis tool integrating the online dataset, phylogenic information, and algorithm yields the separation of OGs and non-Orphan Genes (non-OGs). The inferred OGs are further characterized via gene function study. ******** indicates detailed trait content.

OGs are a fascinating and understudied aspect of genomics, presenting a significant challenge in the field of molecular biology. An OG is defined as a gene that lacks significant sequence similarity to any known genes, consequently resulting in limited functional annotation or information about its biological role [54]. The identification of these genes is important as they may contribute to the evolution of novel adaptations and the regulation of physiological processes [8]. In this review, we will discuss the various methods used to identify OGs, the challenges associated with this process, and their potential biological significance. Comparative genomics (Figure 1) enables the identification of conserved and unique genes across diverse species [2]. Those conserved genes are presumed to possess significant functions, while the unique across species are regarded as potential candidates for OGs [64]. However, this approaches has its limitations, as certain OGs may have undergone rapid evolutionary changes and thus lack conservation, posing challenges for their detection [11]. Alternatively, BLAST, Phylostratigraphy and ORFan-Finder, discussed below, are potential alternatives to overcome all these limitations.

### 3.1. Methods of Orphan Genes Discovery

#### 3.1.1. BLAST

The accurate identification of OGs is an essential prerequisite for understanding the evolutionary and functional roles of these genes in various organisms. BLAST is a widely used tool for aligning sequences and searching for homologous genes across different species [57]. It compares sequences and scores them based on their similarity. BLAST is the method of choice for locating gene homologs and determining their evolutionary relationships [1]. However, it is important to determine if a gene is absent from other lineages or if its absence is due to the limitations of the BLAST method [65]. Several studies have evaluated the performance of BLAST in detecting distant homologs and found it to be effective in this regard [66]. Nevertheless, some genes may have diverged extensively, making them difficult to be detected through BLAST; in which case, a more sensitive method such as Position-Specific Iterated BLAST (PSI-BLAST) can be used [52]. PSI-BLAST builds a profile of the most conserved residues from closely related homologs, enabling the identification of more distant homologs. However, PSI-BLAST requires manual monitoring and may track convergent gene families, limiting its suitability for large-scale investigations [1].

Furthermore, OGs exhibit differences from normal protein-coding genes in terms of gene length, exon count, GC content, and expression level. These distinctions enable their identification using protein features such as employing BLASTp [67]. Recently, machine learning-based approaches have been developed to identify OGs by leveraging protein features between OGs and non-OGs. Those approaches use machine learning, including deep learning, to extract features from raw sequences and identify OGs [68]. For instance, researchers applied a machine learning-based approach to identify risk genes for autism spectrum disorder (ASD) by incorporating spatiotemporal gene expression patterns, gene-level constraint metrics, and other gene variation factors [69]. However, developing an efficient training strategy for models that solely relies on protein sequences and yields reliable results remains a crucial challenge [68]. Collectively, BLAST and PSI-BLAST are valuable tools for identifying OGs; however, there is still room for improvement, and more research is necessary to develop an efficient and dependable strategy for identifying OGs.

#### 3.1.2. Phylostratigraphy

Phylostratigraphy is a bioinformatics technique that utilizes evolutionary information, such as fossil records and molecular data, to determine the evolutionary age of a gene. The application of phylostratigraphy to the study of OGs involves inferring the evolutionary age of a gene and comparing it to the age of the species in which it is found [70]. This approach helps differentiate between genes that have a homolog in a closely related species and genes that have evolved independently in the species of interest. This technique utilizes homology searches and BLAST to estimate a gene’s evolutionary age by comparing it to related species’ proteomes. However, some genes may evolve more quickly and diverge from their homologs, leading to an underestimation of their age when using phylostratigraphy [71]. Therefore, it is important to consider synteny data in phylostratigraphic analysis to accurately determine the evolutionary age of a gene.

Studies using phylostratigraphy have indicated that the rate of de novo gene synthesis is equal to or greater than the rate of gene duplication. For example, the yeast genome is thought to contain hundreds of de novo genes that have emerged throughout *Ascomycota* evolution, and at least nineteen of these genes are specific to *Saccharomyces cerevisiae* (*S. cerevisiae*) [72]. Similarly, it has been estimated that 780 unique genes have evolved in mice since their split from the Brown Norway rat, with half of all young mouse genes believed to be de novo genes [6]. It is important to note that rapid de novo gene synthesis must be accompanied by rapid gene loss to maintain stable gene numbers in a species over time.

Furthermore, a syntenic analysis can be useful for distinguishing between de novo protein-coding and non-coding genes in closely related species, due to their rapid evolutionary change [73]. However, synteny has mostly been used in study-specific investigations or cases where curated genome options are available. Nonetheless, a recently published R package *fagin* provides an enhanced method for analyzing de novo genes [74]. It utilizes an automated and comprehensive analysis of synteny-based phylostratigraphy, allowing for the identification of newly evolved orphan and lineage-specific genes [75].

#### 3.1.3. ORFan-Finder

OGs are also referred to as new genes, lineage-specific genes (LSGs), and taxonomically restricted genes (TRGs), and the origin of these genes is often termed “de novo-created novel genes” [1]. Computational methods and machine learning (ML) techniques are widely used to identify OGs in large genomic datasets. One such tool is ORFan-Finder, which employs various strategies to identify ORFans/OGs, including BLAST searches, Hidden Markov Model (HMM) profiles, and comparative genomics. By analyzing the presence and absence of OGs in different species [76], ORFan-Finder provides insights into the evolution of novel genes. Additionally, ORFan-Finder provides functional annotations and classifications of the identified OGs [76]. This allows researchers to infer the potential functions and roles of the newly discovered genes.

Another notable tool is the SMOTE-ENN-XGBoost model, which utilizes the Synthetic Minority Over-sampling Technique, Edited Nearest Neighbors, and eXtreme Gradient Boosting algorithm for data analysis [77]. Yet, other effective platforms are the BIND (BRAKER-Inferred Directly) and MIND (MAKER-Inferred Directly) systems, which use machine learning to infer gene structure. For instance, BIND and MIND have been found to have the highest overall prediction accuracy in *Arabidopsis thaliana* (Arabidopsis), with BIND recognizing 99% of ancient genes and 68% of annotated OGs [19,78]. Collectively, the study of OGs has been revolutionized by the development of various computational tools and techniques. Additionally, the combination of BLAST and microarray-based genome hybridization methods has proven useful in the study of OGs.

### 3.2. Orphan Genes Databases

The Orphan Gene Databases are an invaluable resource for researchers studying OGs, as those genes have limited sequence similarity to known genes and thus lack extensive functional annotations or information regarding their biological roles. However, detecting OGs can be challenging due to the limited availability of OG-identifying software. The available software may have a restricted database search range or be too complex algorithmically. Therefore, researchers studying OGs often need to collect data from multiple sources. Several databases provide valuable resources for researchers studying the origins, functional aspects, and evolutionary history of OGs in all domains of life.

#### 3.2.1. NCBI

NCBI is an essential resource for researchers studying OGs [6]. It hosts several critical databases for the research community, including GenBank, BioProject, and Taxonomy. GenBank serves as a repository for annotated nucleotide sequence data, containing 2.5 × 10^11^ bases from 2.0 × 10^8^ sequences. BioProject, formerly known as GenomeProject, provides Whole-Genome Sequencing (WGS) data for over 130,000 sequencing projects, representing approximately 20,000 species [79]. These databases are essential resources for researchers working in the fields of genomics and bioinformatics.

NCBI’s Gene database provides information on gene sequence, structure, expression, and function, and links to other relevant databases and resources [1]. In addition to the Gene database, NCBI provides access to several other databases and tools that are relevant to OGs [12]. The RefSeq database provides a comprehensive collection of curated and annotated gene sequences, including those for OGs [80]. The NCBI BLAST tool allows researchers to search for homologs of OGs in other organisms, facilitating the identification of potential functions and evolutionary relationships. The NCBI Gene Expression Omnibus (GEO) provides a repository for gene expression data [12], enabling researchers to explore the expression patterns of OGs and their potential roles in disease and other biological processes.

#### 3.2.2. ORFanID

ORFanID is a graphical web-based search engine that assists users in identifying OGs/TRGs at different taxonomic levels, ranging from species to domain. It runs through the NCBI database search parameters using standard NCBI systematic classifiers. ORFanID processes both protein/amino acid sequences and DNA/nucleotide sequences, providing the taxonomic rank of a gene. It builds its database with the analysis results and allows researchers to mine the data further [81].

ORFanID has demonstrated high accuracy in identifying species-specific OGs. For example, it successfully identified the Arabidopsis *QQS* (*QUA-QUINE STARCH*) gene involved in starch biosynthesis, the *Drosophila melanogaster* (*D. melanogaster*) genes *jeanbaptiste* and *karr* that are crucial for male development, and the *S. cerevisiae* genes *bsc4* and *fyv5* that are associated with DNA repair and vegetative growth [81,82]. Therefore, ORFanID is a valuable tool for researchers studying OGs and TRGs.

#### 3.2.3. POGD

The *Poaceae* Orphan Genes Database (POGD) is a newly developed and user-friendly web interface that aims to provide comprehensive information about OGs in four *Poaceae* species [83]. The POGD offers a wide range of information related to gene descriptions, gene product records, and functional annotations. In addition, the website provides a BLAST and comparative analysis for efficient data extraction of target genes. Using the POGD, the percentage of OGs was calculated in the genomes of *Brachypodium distachyon*, *Oryza sativa*, *Sorghum bicolor*, and *Zea mays*, which were found to be 10.35%, 22.78%, 10.92%, and 31.54%, respectively [83]. This information is handy for understanding the distribution of OGs across different plant species and their potential role in plant evolution. Moreover, the POGD database serves as a repository for unraveling the central functions of OGs and can assist in developing comparative genomics in plant biology. The availability of the POGD will help further studies on the regulation of OGs and their roles in the adaptation and diversification of *Poaceae* species.

#### 3.2.4. TOGD

The wheat (*Triticum*) Orphan Gene Database (TOGD) has been developed to provide researchers with access to various features of OGs in wheat, such as their chromosome location, putative functions, and gene structure. This database also offers a flexible search engine with multiple options, a BLAST tool for exploring homologous sequences, and information on protein characteristics and expression patterns from external databases. Through homology searching against 94 plant species, 993 OGs were identified and characterized [84]. As the first OG database in wheat, TOGD is a valuable bioinformatics platform for functional and evolutionary studies of OGs in *Triticum aestivum* (*T. aestivum*), contributing to wheat breeding, seed production, and the development of comparative genomics in wheat biotechnology.

#### 3.2.5. ORFanage

ORFanage is a database that provides comprehensive information on open reading frames in fully sequenced microbial genomes. This database offers three types of ORFans that can be searched within any subset of genomes, allowing users to identify targets for further genomic and evolutionary research [85]. Accessiblethrough http://www.bioinformatics.buffalo.edu/ORFanage (accessed on 29 May 2023). ORFanage consists of two primary sections: the first section provides information on singleton ORFans, including a list of all the genomes in the database and the percentage of ORFans in each genome. The central section of the database is the ORFan searcher, which allows researchers to choose a subset of genomes to search, with the search results delivered via email. With ORFanage, researchers can study family-specific or species-specific proteins or search for potential horizontally transferred genes among unrelated genomes [85]. This database is an essential resource for identifying exciting targets for future studies.

There are several other databases that provide information on OGs in multiple species. ORFanDB (http://cys.bios.niu.edu/ORFanDB/ accessed on 29 May 2023) is an example of a database with an embedded interactive web application. Users can select a species and narrow their selection based on the strain and OG type using a set of nested tabs [80]. Dfam is another database where OGs are often found within repetitive DNA elements. It provides information on the sequence, structure, function, and evolutionary history of those elements [86]. Ensembl is a comprehensive database that offers information on the genomes of various organisms, including OGs. It includes information on gene sequence, structure, expression, function, and links to other relevant databases and resources. These databases can be helpful resources for researchers investigating OGs in multiple species [77]. UniProt is a database that provides comprehensive information on protein sequences and functional annotations, including those of OGs [87]. Researchers can compare OGs to other known protein sequences, identify functional domains or motifs, and investigate their molecular functions and evolution. OrthoDB is another valuable database for studying OGs, providing information on orthologous groups of proteins across various species [88]. Researchers can infer the evolutionary history and functional significance of OGs by comparing them to orthologs from related species. OrthoDB also provides information on gene expression and functional annotations for many species, which helps study OGs in the context of their biological processes [88].

### 3.3. Screening of OGs

Screening for OGs can be challenging due to their unique characteristics, and different screening methods may encounter different problems.

Comparative genomics, facilitated by tools such as BLAST, is one of the most common methods used to identify OGs [1,57]. This method involves comparing the genome sequences of different species to identify the genes present in one species but absent in others. However, comparative genomics may miss highly divergent OGs that cannot be detected using sequence similarity searches. In addition, incomplete or inaccurate genome assemblies can result in the omission of OGs, and some OGs may be misannotated as non-coding regions or pseudogenes [2]. Transcriptome sequencing is another approach used to identify OGs that are actively transcribed [89]. However, this method also has limitations. For example, low expression levels of OGs may make them difficult to detect [67]. Moreover, different isoforms of the same gene can be misannotated as separate OGs. Proteomics/omics [90]-based approaches are extensively employed to identify OGs, with a primary focus on the proteins they express rather than their DNA or RNA sequences. However, proteomics can also encounter several challenges. For example, the low abundance of OG products in the proteome can hinder their detection [90]. Post-translational modifications or alternative splicing can further complicate the identification of OG products [91]. Alternatively, functional screens, such as CRISPR/Cas and Y2H systems, involve experimental manipulation of gene expression or protein activity to identify the functions of OGs [92,93,94]. Although the approach can be used to identify the functions of OGs, the phenotypic effects of some OGs may be subtle or difficult to detect. Furthermore, the functions of OGs may be highly context-dependent and may not be revealed under all experimental conditions.

In addition to the specific challenges faced by these screening methods, several conditions can affect OG screening. For example, the quality of genome or transcriptome assemblycan significantly impact the detection of OGs [89]. Sequencing errors or gaps can also affect identifying OGs [95]. The genetic diversity of the species being studied can pose challenges in OG screening, particularly in highly diverse or poorly characterized species. Finally, additional factors, including the evolutionary age, functional divergence, and tissue-specific or developmental stage-specific expression can also affect their detection and characterization [50,77].

To overcome these challenges, researchers have developed a range of approaches and techniques to identify and study OGs. One approach is the combination of multiple screening methods to increase the sensitivity and specificity of detecting OGs. For example, integrating transcriptome sequencing with proteomics or functional genomics offers a more comprehensive understanding of OG expression, structure, and function [96]. In addition, developing more sensitive and specific algorithms and tools for analyzing genomic, transcriptomic, and proteomic data can improve OG detection and annotation accuracy and reliability [13]. Another approach is to use a phylogenetic analysis to infer the evolutionary history and function of OGs [6]. This approach aids in identifying the emergence and diversification of OGs and their potential roles in evolutionary innovation and adaptation [58]. Machine learning (ML) approaches are also being developed to predict OG functions based on their sequence features and structural properties. For example, ML models can be trained on large-scale genomic and functional data to predict the functions of OGs based on their sequence and structural features [69]. This approach helps prioritize OGs for experimental validation and provides insights into their functional roles and mechanisms.

Overall, screening OGs is an exciting and rapidly evolving area of research that has the potential to reveal important insights in gene evolution and function and their potential implications for disease. As our understanding of OGs grows, new screening methods and analytical tools will undoubtedly emerge, further advancing our understanding of these enigmatic genes.

## 4. Functional Characterization

It is often assumed that newly evolved genes are not essential for survival; after all, organisms appear to be able to function without them. While the function of the majority of OGs remains unknown, and they may lack recognized folds, functional motifs, and domains, there is enough evidence of their ubiquitous functionality.

### 4.1. Characterization Based on Functionality of OGs

**Figure 2:**
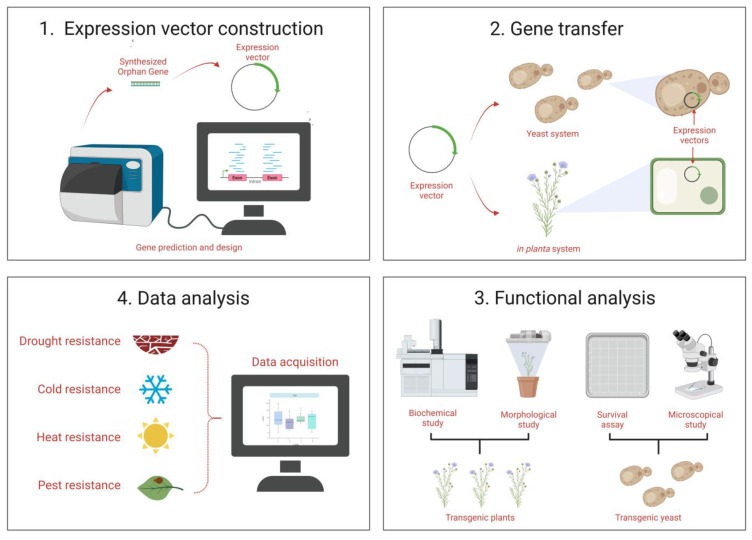
Pipeline of Orphan Genes (OGs) characterization. The inferred OGs are modified to be inserted into gene expression constructs for subsequent use in heterologous hosts, such as yeast or in planta systems. After transformation and selection, stable transgenics can be utilized in a variety of investigations. The potential function can be inferred based on data analysis of the functional studies. This figure was created using BioRender (https://biorender.com/accessed on 28 May 2023).

OGs were first discovered in the yeast genome sequencing project in 1996 [12,97]. They were found to constitute up to 26% of the yeast genome, but it was believed that the number of OGs would likely increase as more genomes were sequenced [98]. With the advancements in sequencing technologies, the number of sequenced genomes has been increasing, leading to the discovery of new OGs (Figure 2). This led to the conclusion that OGs can be found in almost every genome with their specific roles in various biological processes, including metabolism, immunomodulation, stress biology, and other species-specific adaptive processes [8,28,50].

Although several plants encode OGs that have been demonstrated to be necessary for survival under certain situations, none have been reported to be embryo-lethal if they are disrupted [17,99]. It has been observed that purifying selection is prevalent in old genes while younger genes show a higher occurrence of positive selection, suggesting the functional significance of OGs [100]. Several investigations in plants have provided evidence for the role of OGs in modulating carbon or nitrogen metabolism (Table 1). For instance, the QQS in tobacco was found to induce the activity of RubisCO (Ribulose-1,5-bisphosphate carboxylase/oxygenase), an enzyme critical for the initial step of carbon fixation. QQS also directly interacts with *Solanum tuberosum* NF-YC4 (StNF-YC4) [5]. These findings collectively indicate that QQS plays a pivotal role in modulating carbon and nitrogen patterns in plants, further highlighting the potential involvement of OGs in central regulatory networks. Additionally, other OGs, such as *TaFROG* (*Triticum aestivum Fusarium Resistance Orphan Gene*) and *SNF1α* (*sucrose-nonfermenting 1α*), have been implicated in regulating energy homeostasis and sugar metabolism [26]. In *Brassica* rapa, the overexpression of multiple OGs was shown to mediate carbon metabolism, with *Br*OG1-overexpression in Arabidopsis specifically involved in the suppression of the *sucrose synthase* (*SUS*) at the RNA level. Another OG, *BR1* was found to be a novel regulator of flowering time, as its loss resulted in delayed inflorescence development in Arabidopsis [101].

Moreover, OGs have also been shown to play a role in stress resistance and immune regulation [28,64,102]. For instance, *AtQQS* in Arabidopsis and soybean confers resistance against pathogens and pests, and *TaFROG* enhances resistance against the mycotoxigenic fungus *Fusarium graminearum* (*F. graminearum*) [26]. The OG *Xa7* (*Avirulence Xanthomonas resistance 7*) protects the sucrose efflux transporter SWEET14 (Sugars Will Eventually Be Exported Transporter 14) in rice against *Xanthomonas oryzae pv. oryzae*-induced cell death, suggesting a role for OGs in immune responses related to sugar metabolism in plants [103]. OGs have been shown to mitigate hormonal signaling pathways to confer stress resistance in plants. For example, the rice-specific OG *OsDR10* (*Oryza sativa defense-responsive gene 10*) was found to be a negative regulator against *Xanthomonas oryzae* pv. *oryzae*-mediated bacterial blight [104]. The suppression of *OsDR10* resulted in increased levels of naturally occurring salicylic acid (SA), a reduction in jasmonic acid (JA), and the altered expression of multiple resistance (*R*) genes, leading to enhanced disease-resistance functions in rice [104,105]. Pathogens employ effector molecules to induce effector-triggered susceptibility (ETS), a strategy by which they overcome the immune response of the host. Such an ability is possibly achieved through the targeting of the central signaling hub [106,107]. As SA is known to play a central role in defense signaling during pathogen attacks and in establishing resistance [105], it is possible that some OGs act as stress-responsive genes, blocking the hormonal signaling pathway and inducing susceptibility. Additionally, plant OGs interplay with multiple regulators to modulate their function. As an example, SnRK1 (Sucrose non-fermenting-1-related protein kinase 1) in wheat interacts with the *Ta*FROG to confer resistance against *F. graminearum* [26,34].

As sessile organisms, plants encounter multiple stresses, and there is a growing body of evidence suggesting the involvement of OGs in abiotic stressors and hormone-signaling pathways [99,108]. For instance, several OGs from a *Coffea arabica* cultivar that is resilient toward drought have been implicated to contribute to the abscisic acid (ABA) pathway [33], which serves a crucial signaling transduction pathway in plant response to drought conditions [109]. Moreover, the transcriptome profiling of the moss *Physcomitrella patens* (*P. patens*) unveiled a prominent role of OGs in early cold stress responses. Interestingly, those moss- secreted OGs were found through deep sequencing to be highly enriched in endosymbiotic bacteria *Buchnera aphidicola*, which play a role in the aphid response to drought stress. Furthermore, transgenic Arabidopsis expressing the *P. patens*-specific OG *PpARDT* (*ABA-responsive drought tolerance*) exhibited enhanced drought resistance, potentially achieved by regulating the ABA signaling [23]. The SnRK1 function is activated by ABA, which in turn triggers extensive transcriptional and metabolic reprogramming for energy metabolism [110].

Several studies have explored the role of OGs in various biological processes, though the complete mechanism of their action remains unknown. It has been well established that OGs are involved in primary substance metabolism, the response to biotic and abiotic stress, and the formation of species-specific traits [2]. However, recent studies have suggested that the significant proportion of ORFs found in humans might be mis-predicted due to their small size and low sequence conservation across species [111]. Despite the high degree of sequence divergence, the subgroup of primate OGs that generate experimentally functional proteins is comparable to the remaining primate OGs [2]. The orphan nuclear receptor NR2E3 is a direct transcriptional target of the major basic motif leucine zipper transcription factor, which determines the fate of rod versus cone photoreceptor cells in the human retina [112]. Dysfunctional NR2E3 leads to increased S-cones and rod degeneration in humans, as well as retinal degeneration in *rd7* (*Retinal degeneration* 7) mutant animals. On the other hand, ectopic expression of *Nr2e3* in the cone-only Nrl/retina results in rod-like cells without visual functions [113]. Transgenic mice experiments have demonstrated that *Nr2e3* can restore rod photoreceptor functions while suppressing cone gene production when produced under the control of the *Crx* (*Cone-Rod Homeobox*) promoter. Furthermore, *Nr2e3* expression in photoreceptor precursors committed to becoming rods (controlled by the *Nrl* promoter) was able to completely reverse the retinal phenotype of *rd7* mice [114]. Additionally, another OG, *FLJ33706*, has also been be associated with the potential pathogenesis of Alzheimer’s disease in humans [115]. Orphan G-protein-coupled receptors (oGPCRs) are also a focus of research due to their potential as therapeutic targets. Despite the limited knowledge about their ligands and linkage to cellular signaling mechanisms, oGPCRs are expressed at lower levels in the brain, and their function remains unknown. Expression profiling is essential to determining their role in brain function and illness; however, the currently available databases provide limited information in this regard [116]. Due to their cell surface accessibility, all GPCRs, including oGPCRs, are attractive targets of drug development, and modern techniques such as allostery, bias, or structure-based docking approaches can be employed to develop novel therapeutics [117,118]. However, the function of oGPCRs in the brain remains unclear, and these receptors are understudied [119]. Plasmalogens, a type of glycerophospholipids characterized by a signature sn-1 vinyl ether bond, have been linked to membrane organization, signaling, and antioxidant functions in mammals and microbes. The human enzyme coding gene *TMEM189* (*transmembrane protein 189*) and its bacterial homolog *CarF* have plasmanylethanolamine desaturase activity, which is required for the production of vinyl ether bonds. Plasmalogens contribute to photooxidative stress sensing through singlet oxygen, and *CarF* promotes light-induced carotenogenesis in a bacterium *Myxococcus xanthus* [120]. The discovery of the human plasmanylethanolamine desaturase will spur further research into its biogenesis, functions, and involvement in the disease of plasmalogens [120]. Additionally, cytochrome P450 2S1 (CYP2S1) is an orphan cytochrome P450 enzyme (CYP) predominantly expressed in extrahepatic tissues and is inducible by dioxin. Although extra-hepatic CYPs play a minor role in drug metabolism, they are crucial for causing in situ toxicity in tissues with greater expression [121].

The adaptability of fungi in various ecological niches relies on their response to environmental changes. One key component enabling this adaptability is the fungal secretome, which is composed of proteins involved in the breakdown of organic materials [122]. These proteins include proteases, lipases, carbohydrate-active enzymes (CAZymes), hydrophobins, and small-secreted proteins (SSPs) [123]. SSPs are proteins with a signal peptide and a sequence of fewer than 300 amino acids, comprising 40 to 60% of the fungal kingdom’s secretome. Many SSPs are encoded by OGs and are particularly important in fungi that interact with living hosts. For instance, cysteine-rich “effectors” among SSPs play a crucial role in reducing host defense responses and altering host physiology during infection [124,125]. In recent years, several genes that are important for various stages of infection in *F. graminearum* have been identified. For example, the *FGL1* and *FgNahG* effector genes have been found to play a significant role [126]. *FGL1* encodes a secreted lipase that decreases immunity-related callose synthesis during wheat head infection, while the importance of multiple other OGs in the *F. graminearum* genome is yet to be elucidated [127]. The *Osp24* (*orphan secretory proteins 24*) gene in *F. graminearum* encodes a cytoplasmic effector that targets *Ta*SnRK1α for degradation [64].

In another study, the transcriptomics of whole-genome cold stress in moss *P. patens* and Arabidopsis revealed that these organisms respond to early stress signals by initiating the cold acclimation process through the expression of genes associated with transcription-associated proteins [23]. Furthermore, genome sequencing of *S. cerevisiae* provides new insights into the expression and function of genes, as well as the evolution of eukaryote genomes. Proteome comparisons between yeast and worms revealed that the core metabolic process genes remain unchanged in their function, but OGs in yeast are rapidly evolving in their proteome fractions.

### 4.2. Genetic Basis and Morphological Level

Functional annotation of OGs has revealed their crucial role in the development of male gametophytes [45]. Two bread wheat OGs specific to *Poaceae* family, namely *male sterility 1* (*Ms1*) and *male sterility 2* (*Ms2*), confer important traits in wheat breeding, owing to their vital roles in pollen biology and male fertility [32,128]. Additionally, the expression levels of OGs in the flowers of the *Cucurbitaceae* family were markedly higher in contrast with the rest of the parts [8], suggesting a spatial role in modulating the various regulatory pathways associated with male fertility. Moreover, the *GS9* (*Grain Shape Gene on Chromosome 9*) gene in rice participates in inflorescence formation and influences the grain morphology and visual traits. It interacts and colocalizes with *OsOFP14*, an OVATE family protein in the nucleus, thereby regulating the fruit shape [129]. Despite an incomplete understanding of the underlying mechanism, several studies have provided strong evidence for OGs’ involvement in primary substance metabolism, stress responses, and the formation of species-specific traits (Table 1).

Mycorrhizal fungi form mutualistic relationships with plants to facilitate nutrient acquisition, and these symbioses have been observed in multiple lineages of Mucoromycotina, Ascomycota, and Basidiomycota [125]. Despite the frequent emergence of this guild in nature, the genetic traits underlying ectomycorrhizal lifestyle shifts are irreversible evolutionary transitions, and future research should focus on factors that predispose certain organisms to form these symbioses [124]. In 2009, a study using phylostratigraphy on representative genomes of prokaryotes, plants, animals, and fungi such as Basidiomycota, major lineages of Ascomycota, and *Chaetomium globosum* (a species closely related to *N. crassa*), successfully identified 2219 OGs in *N. crassa* [130]. Among these OGs, several are allorecognition loci commonly referred to as het (heterokaryon incompatibility) or vic (vegetative incompatibility) genes. These genes regulate allorecognition during vegetative growth and play a crucial role in determining compatibility between individuals, allowing only those with compatibility at all het-loci to fuse and expand their colonies [131].

Furthermore, the study of basal metazoans has expanded our understanding of the functions of genes, with evidence suggesting that their involvement in crucial adaptive processes is specific to each species. For example, research in Hydra has revealed that TRGs played a crucial role in the development of novel traits specific to their phylum [132]. Genomics and transcriptomic sequencing have provided evidence of a gradual evolution of the molecular mechanisms underlying development, resulting in an intriguing evolutionary paradox attributed to the remarkable conservation observed in signal transduction pathways [133]. This paradox can be explained by the evolution of regulatory genes, which are present throughout the animal kingdom and contribute to morphological differences among species by utilizing similar components differentially [59] (Figure 2). In salamanders, the *Prod1* gene can regulate limb regeneration by determining the direction of limb growth [134]. In Drosophila, six genes were found to be essential for organismal fitness and metamorphosis [135]. In ants and other members of phylum Hymenoptera, OGs have been implicated in social evolution [136]. Collectively, OGs hold the potential to reveal the mechanisms of the origin of protein structural domains, which is of great significance as they offer opportunities for the creation of new proteins. However, their long-term significance for evolution remains unknown. Future research should focus on understanding the function and evolution of OGs in fungi and their impact on interactions with host organisms.

## 5. Role of OGs in the Prokaryotic and Viral World

OGs in prokaryotes are a subject of great interest in current molecular biology research. The discovery of OGs has been facilitated by the availability of genomic data from a diverse range of prokaryotic species. It has been well studied that OGs often contribute to the acquisition of novel traits and play crucial roles in facilitating the adaptation of their host organisms to dynamic environments [134]. Previous studies have observed the extensive presence of OGs in bacterial genomic islands (GIs) [137], which are clusters of horizontally transmitted genes, including virulence factors (VFs). These GIs, also known as pathogenicity islands (PAIs), possess the capability of transforming non-pathogenic bacteria into pathogens [80]. PAIs tend to contain a higher proportion of VF genes compared to other regions of the genome [138]. Another study identified 39% of OGs in genes clustered with unusual base compositions, which are believed to be indicative of horizontal transfer from bacteria or viruses, in 119 prokaryotic genomes [51]. Many of the unique genes identified in PAIs or prophages are lineage-specific OGs, which may contribute to the pathogenicity of the bacteria [139]. A recent study has illustrated this by characterizing the function of an OG named *neat* (*nomadically evolved acyltransferase*) in extraintestinal pathogenic (*ExPEC*) *Escherichia coli*, which indicates its pivotal role in the virulence of *ExPEC* in zebrafish embryos [46]. Despite the molecular biology community’s tendency to focus more on conserved genes, taxonomically restricted OGs are likely to be of greater significance in terms of the emergence of species-specific traits. For example, they are thought to be key contributors to the ability of pathogens to infect their hosts.

The field of viral genomics has experienced a resurgence in recent times, owing to the recognition of viruses, particularly phages, as significant contributors to evolution. To date, over a thousand complete viral genome sequences, including hundreds of phages, have been made publicly available [140]. Studies of phage genomes have demonstrated that HGT, which is also a proposed model for OG origination, has had a significant impact on viral genome evolution [141]. HGT occurs predominantly between phages within the same host cell or with prophages that are present in the host genome [138]. The phages can exchange genes through integration with prophages and recombination by exchanging particular genes with the host, and it was recently observed in Cyanobacteria and Cyanophages through horizontal transfer to phages from the host, suggesting that they’ve shared the same evolutionary pathway with OGs [142]. Additionally, in whitefly, there is evidence of differential regulations of OGs involved in various processes, including glucose transport, the uric acid pathway, metabolic pathways, signal transduction, immune modulation, and potential receptor functions [143]. A recent study examined the expression of these genes in whiteflies feeding on plants infected with *Tomato chlorosis virus* (*ToCV*) compared to those feeding on uninfected plants. The results showed differences in gene expression between the two groups, providing insights into the potential role of these genes in the interaction of whiteflies with *ToCV*-infected host plants [143].

Moreover, the percentage of OGs varies among species, even among those with fully sequenced genomes. However, the annotation of hypothetical proteins has reduced the number of OGs with unknown functions, as recorded in various databases [141]. Approximately one-third of OGs are found in virus genomes, particularly in prokaryotes [140]. Viral OGs tend to have a lower GC content and shorter lengths compared to non-OGs. However, this lower GC content is only statistically significant in a minority of viruses. Phage OGs and non-OGs have been found to have a similar distribution against the genome of prokaryotic organisms, with roughly half of the phage ORFs having homologs in prokaryotes [144,145]. Furthermore, comparative analysis has revealed that the genomes of newly discovered viruses often contain a high proportion of orphan- and taxon-specific proteins that lack recognizable homologs due to the rapid evolution of viral proteins. The identification of homologs can be facilitated through a BLAST similarity sequence analysis (Figure 1) [57]. Powerful tools have been developed in recent years to detect specific homologs among the top quarter of proteins with the best properties for annotating the genomes of RNA viruses, including the detection of orphan proteins [146]. Several viruses such as *chronic bee paralysis viruses* and *alphaviruses*, such as *cile*, *higre*, *nege*, and *bluner* viruses, have been reported to have a high proportion of orphan proteins [146,147]. These findings suggest that the evolution of viruses often results in a high number of orphan- and taxon-specific proteins that lack recognizable homologs.

In conclusion, the function and origin of OGs remain largely unknown and require further elucidation in various organisms descended from common ancestral proteins through adaptive variation and duplication. To gain a better understanding of their mysterious origins, additional studies need to be conducted.

## 6. Role of Dark Transcriptomics in OG Evolution

For decades, it has been believed that new genes coding for proteins emerged primarily through mutations in existing genes. However, recent research has revealed the existence of OGs, which code for proteins that are unrelated to those found in other species. We now know that OGs have been identified in nearly every species, and that they play a critical part in major growth and developmental pathways through interacting with conserved transcription factors, central regulators, and receptors [5,20,148]. OGs, also known as TRGs, are a component of the genome with largely unknown functions and ancestry [4,133]. With the redefinition of these genes as species-level TRGs, researchers have been studying these mysterious genes to uncover their functions and regulation. One field that has advanced the understanding of these genes is dark transcriptomics, which focuses on identifying transcripts that are not translated into protein [76,78]. High-throughput sequencing techniques, such as RNA sequencing (RNA-seq), are used to identify transcripts that are not associated with known protein-coding genes [149,150,151]. By comparing the transcriptome to the annotated genome, researchers can identify novel transcripts that do not correspond to known protein-coding genes [76]. This approach has provided new insights into the function and regulation of OGs, including those that are transcribed and regulated despite not producing a protein [78]. Dark transcriptomics have revealed that some OGs are involved in regulating the expression of other genes. Additionally, some OGs are transcribed into long non-coding RNAs (lncRNAs), which play important roles in cellular processes such as gene regulation, chromatin remodeling, and mRNA stability [76]. The identification of alternative splicing events, which can result in the production of different transcript variants from a single gene, has also shed light on the role of OGs in the transcriptome [152]. Overall, dark transcriptomics have greatly impacted our understanding of the role of OGs in the transcriptome. The identification of novel transcripts and alternative splicing events has opened up new avenues for research and provided new insights into the function and regulation of these fascinating genes. Furthermore, the identification of transcripts from OGs holds promise for revealing new targets for therapeutic intervention and could potentially lead to the development of innovative treatments for various diseases.

## 7. Future Directions for Orphan Genes Research

Due to their lack of a recognizable function, the discovery and characterization of OGs have been a focus of genetic research for decades. The study of OGs is crucial to understanding the functional diversity of the genome and the evolution of complex traits. As genomic sequencing technology continues to improve, the identification and characterization of OGs are becoming more straightforward. In this section, we outline several areas of research that hold promise for advancing our understanding of OGs.

### 7.1. Functionality Prediction

The development of computational methods for predicting the function of OGs is one of the most promising areas of research. ML algorithms and sequence comparison tools can be used to identify potential functional elements within OGs, such as protein domains and regulatory motifs. Experimental validation is then needed to confirm these predictions.

### 7.2. Comparative Genomics

The comparison of genomes from different species can also provide insights into the function of OGs. For example, the presence of an OG in several closely related species may suggest that it has a conserved function, even if its exact role is unknown. Genes conserved across multiple species can be prioritized for functional studies, as those are likely to have critical roles.

### 7.3. Tissue-Specific Expression

The tissue-specific expression of OGs can also provide clues about their function. For example, the expression of an OG in a specific tissue suggests that it may be involved in the development or maintenance of that tissue. This approach can be combined with other functional genomics techniques, such as transcriptomics, to identify co-regulated genes with potential similar functions.

### 7.4. Gene Expression and Knockdown Experiments

Gene expression and knockdown experiments are powerful tools for investigating the function of OGs. By comparing the gene expression profiles of cells or tissues with and without an active OG, researchers can identify genes that are regulated by the OG and gain insights into their role in cellular processes. Similarly, knockdown approaches can be used to determine the effect of reducing the expression of an OG on cellular processes and phenotypes. For instance, the functionality of an orphan protein may be transferable to ectopic species using cutting-edge technologies such as CRISPR/Cas [92,153] or Agrobacterium-mediated transformation [154], while yeast heterologous systems [155] along with other techniques can be employed for the cross-species/kingdom characterization of these OGs (Figure 2).

### 7.5. Evolutionary History

Finally, the evolutionary history of OGs can shed light on their function. For example, the presence of an OG in several distantly related species suggests that it may have an ancient function that has been conserved throughout evolution. On the other hand, the rapid divergence of an OG in multiple lineages indicates that it may have acquired distinct functions in different species.

## 8. Concluding Remarks

In conclusion, the study of OGs has made significant progress in recent years, thanks to advancements in tools and technologies. However, much work remains to be conducted to fully understand the function of these genes and the roles they play in cellular processes and organismal biology. The research areas outlined above represent some of the most promising avenues for future work in this field, and they hold the potential to significantly advance our understanding of the functional diversity of the genome.

To summarize, the discovery and characterization of OGs are essential aspects of genetic research that can contribute to our understanding of the functional diversity of the genome and the evolution of complex traits. Future directions for this field include the development of computational methods for predicting gene function, the utilization of comparative genomics and tissue-specific expression studies, gene expression and knockdown experiments, and investigations into the evolutionary history of OGs. These areas of research hold tremendous potential for advancing our understanding of OGs and their significance in cellular processes and organismal biology.

## Figures and Tables

**Table 1:** Different OGs identified in multiple hosts with their functions.

Orphan Gene	Corresponding Host	Function/s	Reference
■ *AtQQS*	*Arabidopsis thaliana*	Reduces susceptibility to pathogens and pests	[20]
■ *Dauerless*	Nematodes	Inhibitor of Dauer development	[3]
■ *Tetherin*	Vertebrates	Antiviral activity	[21]
■ *BroGs*	*Brassica rapa*	Primary metabolism	[22]
■ *PpARDT*	*Physcomitrium patens*	Drought tolerance	[23]
■ *QQS*	Soybean	Modulates carbon and nitrogen allocation	[24]
■ *Xa7*	*Oryza sativa*	Executor resistance gene against *Xanthomonas oryzae* pv. *oryzae* (*Xoo*)	[25]
■ *TaFROG*	Wheat	Biotic stress resistance	[26]
■ *CcUNK8*	*C. canephora*	Protects plants against drought	[27]
■ *Xio1*	*Oryza*	Triggers enhance resistance to *Xanthomonas oryzae* pv. *oryzae* in rice	[28]
■ *Tssor-3 and Tssor-4*	*Plutella xylostella*	Role in male fertility in *P. xylostella*	[29]
■ *MoSPC1*, *MoSPC2*, *MoSPC3 and MoSPC7*	*Magnaporthe oryzae*	Species-specific adaptive processes	[30]
■ *CcUnk*	Coffee	Involved in abiotic and biotic stress responses	[31]
■ *Ms2*	Wheat	For recurrent selection and hybrid seed production in wheat	[32]
■ *IAPAR59*	Coffee	Drought tolerance in coffee	[33]
■ *TaSnRK1αs*	Wheat	Contributes positively to wheat tolerance of DON	[34]
■ *F58H7.5*	*C. elegans*	Involved as RNA intermediate	[35]
■ *Pf-5*	*Pseudomonas fluorescens*	Produces six secondary metabolites	[36]
■ *nog1*	*Escherichia coli*	Involved in *E. coli*’s central metabolism	[14]
■ *YbjN*	*Escherichia coli*	Regulating bacterial multicellular behavior and metabolism	[37]
■ *htgA*	*Escherichia coli*, *Shigella spp.*	Responsible for lineage-specific adaptations	[38]
■ *YDR393w* (*SHE9*)	*Saccharomyces cerevisiae*	Compromises cell growth	[39]
■ *YgaV*	*Escherichia coli*	Auto-regulated and TBT-inducible repressor	[40]
■ *MXAN_4468*	*Myxococcus*	Negative regulatory role in *M. xanthus*	[41]
■ *PKS-NRPS*	*Aspergillus terreus*	Monitoring conditions for secondary metabolite production	[42]
■ *Gpr49*	Human	New therapeutic target in the treatment of HCC	[43]
■ *KIR2DS3*	Human	Contributes to the diversity of KIR haplotypes	[44]
■ *C19orf12*	Human	Causes a distinct clinical subtype of neurodegeneration with brain iron accumulation	[45]
■ *Neat*	*Escherichia coli*	Key role in the virulence of ExPEC in zebrafish embryos	[46]
■ *AtMO1-4*, *Glycine max src2*	*Arabidopsis thaliana*	Unknown function, showing tissue-specific expression	[47]
■ *AtPCMP*	*Arabidopsis thaliana*	Codes for a novel protein family unique to plants	[48]
■ *ATII LCL*	Atlantis II Red Sea brine pool	Confers antibiotic and anticancer effects	[16]
■ *ritR*	*Streptococcus pneumoniae*	Maintains iron homeostasis in *S. pneumonia*	[49]

## Data Availability

Not applicable.
